# Estimating Wastewater Impacts from Fracking

**DOI:** 10.1289/ehp.121-a117

**Published:** 2013-04-01

**Authors:** Charles W. Schmidt

**Affiliations:** Charles W. Schmidt, MS, an award-winning science writer from Portland, ME, has written for *Discover Magazine, Science,* and *Nature Medicine*.

Wastewater produced by hydraulic fracturing (“fracking”) for natural gas in the Marcellus Shale is already overwhelming disposal options and will continue to do so as gas development increases, according to newly published research.^1^ The investigation did not evaluate environmental consequences of the wastewater. But lead author Brian Lutz, an assistant professor in the Kent State University Department of Biology, says fracking wastewater could have a range of environmental and health impacts if not managed correctly. The analysis was limited to Pennsylvania, which along with West Virginia dominates Marcellus shale gas production today.^2^

During fracking, a fluid mixture pumped deep underground fractures the rock to liberate trapped natural gas, which then rises through the well to the surface. Fracking fluids amount to between 3 and 7 million gallons of water per well,^3^ mixed with sand and a complex chemical mixture that can include naphthalene, formaldehyde, and a variety of volatile organic compounds, among other substances.^4^

Since the 1850s, natural gas has been extracted from a number of relatively shallow formations. But advances in horizontal fracking have enabled the development of shale gas deposits, most of which are more than a mile underground in the Marcellus and even deeper in other formations such as the Woodford Shale in Oklahoma.^5^ In 2010 shale gas contributed 23% of domestic natural gas production, compared with 2% in 2000.^1^ And of that, about 10% came from the Marcellus, which in recent months has become the biggest producer of shale gas in the United States.^2^ Correspondingly, the amount of wastewater generated by fracking in the Marcellus rose nearly sixfold between 2004 and 2011, such that it increasingly dwarfs amounts produced by conventional gas drilling—even though conventional gas production generates 65% more wastewater per unit of recovered gas than fracking does.^1^

According to Kevin Sunday, the deputy press secretary with the Pennsylvania Department of Environmental Protection (DEP), gas producers in Pennsylvania traditionally sent their wastewater to municipal water treatment plants for purification and then discharge into rivers. But with the shift to nonconventional production, the wastewater—which is enriched with heavy metals, radionuclides, and salts liberated from the shale rock below^1^—became harder to deal with. Removing dissolved salts, in particular, requires expensive distillation or reverse osmosis.

Citing these environmental concerns, DEP secretary Michael Krancer called on the Marcellus Shale industry to cease wastewater delivery to municipal sewage plants in April 2011.^6^ According to Sunday, the industry immediately complied and accelerated what was already an ongoing shift of delivering the wastes to private industrial treatment facilities that were better able to precipitate metals and filter out suspended solids. When the state had earlier imposed a more stringent discharge standard for treated wastewater of 500 mg/L of total dissolved solids, drillers had found it more cost-effective to invest in centralized and mobile wastewater treatment for recycling and reuse in fracking operations than to discharge into the environment. Today, Sunday says, “Recycling has never been higher. About seventy percent of flowback water gets reused, with some operators at a hundred percent.”

But Lutz says the decline in wastewater discharge was also met with a significant increase in the amount of wastewater trucked to Ohio for disposal via underground injection, from roughly 26 million gallons in 2010 to 106 million gallons in 2011. Most of Pennsylvania’s geology is not amenable to this practice, he says. Roughly a dozen small earthquakes linked to underground wastewater disposal at facilities near Youngstown, Ohio, between March and December 2011 will likely constrain that disposal option in the future, he adds.

“There was an eleven-month window last year of no new permits being issued for underground injection, but this was an unofficial moratorium, not specified in the documents,” Lutz says. “And while new permits are now just being issued, they have slowed the rate of permit approvals.” Moreover, Ohio regulators now require far lengthier and more thorough review of geological records, Lutz says, which serves to make underground disposal much more expensive than it used to be. The bottom line, he emphasizes, is that the amount of wastewater being generated is going up exponentially at the same time that opportunities for managing it are becoming more limited. “And this creates more opportunities for human exposure,” he says.

According to Jim Erb, a private consultant and former director of Pennsylvania’s Bureau of Oil and Gas Management, stakeholders are now debating future disposal options that range from increases in wastewater recycling to the possibility of more injection wells near Pennsylvania’s border with Ohio, where the geology is more suitable.

Meanwhile, researchers are trying to get a better handle on potential health effects from contact with the wastewater, in addition to other exposures linked to fracking operations. According to Trevor Penning, director of the University of Pennsylvania (UPENN) Center of Excellence in Environmental Toxicology, most reports of health problems linked to fracking thus far are anecdotal. “At the end of the day, we need to show some kind of association to demonstrate causality,” he says. “What we are missing is exposure data on individuals who feel their health is being impacted by living in communities where fracking is happening.”

Among other efforts to examine public health impacts of fracking,^7,8,9^ The UPENN center and nine other Environmental Health Core Centers funded by the National Institute of Environmental Health Sciences are collaborating with managed care companies to analyze community health outcomes data and water quality measurements, comparing localities where fracking is occurring with those where it’s not. According to Penning, the top priority is to identify specific chemical hazards in both air and water for human health risk assessment.

Penning points out that chemicals in fracking fluids—although they make up only a small percentage (0.5–2.0%) of the total aqueous volume—are present in large amounts given how much water goes into a single well. “This is a complex mixtures problem,” he says. “And a major concern is that wastewater will leak from holding ponds into groundwater and surface water supplies, where human exposures are possible.”
